# Sex differences in long-term mortality among acute myocardial infarction patients: Results from the ISAR-RISK and ART studies

**DOI:** 10.1371/journal.pone.0186783

**Published:** 2017-10-20

**Authors:** Romy Ubrich, Petra Barthel, Bernhard Haller, Katerina Hnatkova, Katharina Maria Huster, Alexander Steger, Alexander Müller, Marek Malik, Georg Schmidt

**Affiliations:** 1 Innere Medizin I, Klinikum rechts der Isar, Technische Universität München, Munich, Germany; 2 Institut für Medizinische Statistik und Epidemiologie, Technische Universität München, Munich, Germany; 3 National Heart and Lung Institute, Imperial College, London, England; 4 DZHK (German Centre for Cardiovascular Research), Partner site Munich Heart Alliance, Munich, Germany; University of Bologna, ITALY

## Abstract

**Background:**

Mortality rates in females who survived acute myocardial infarction (AMI) exceed those in males. Differences between sexes in age, cardiovascular risk factors and revascularization therapy have been proposed as possible reasons.

**Objective:**

To select sets of female and male patients comparable in respect of relevant risk factors in order to compare the sex-specific risk in a systematic manner.

**Methods:**

Data of the ISAR-RISK and ART studies were investigated. Patients were enrolled between 1996 and 2005 and suffered from AMI within 4 weeks prior to enrolment. Patients of each sex were selected with 1:1 equivalent age, previous AMI history, sinus-rhythm presence, hypertension, diabetes mellitus, smoking status, left ventricular ejection fraction (LVEF), and revascularization therapy. Survival times were compared between sex groups in the whole study cohort and in the matched cohort.

**Results:**

Of 3840 consecutive AMI survivors, 994 (25.9%) were females and 2846 (74.1%) were males. Females were older and suffered more frequently from hypertension and diabetes mellitus. In the whole cohort, females showed an increased mortality with a hazard ratio (HR) of 1.54 compared to males (p<0.0001). The matched cohort comprised 802 patients of each sex and revealed a trend towards poorer survival in females (HR for female sex 1.14; p = 0.359). However, significant mortality differences with a higher risk in matched females was observed during the first year after AMI (HR = 1.61; p = 0.045) but not during the subsequent years.

**Conclusion:**

Matched sub-groups of post-AMI patients showed a comparable long-term mortality. However, a female excess mortality remained during first year after AMI and cannot be explained by differences in age, cardiovascular risk factors, and modes of acute treatment. Other causal factors, including clinical as well as psychological and social aspects, need to be considered. Female post-AMI patients should be followed more actively particularly during the first year after AMI.

## Introduction

Survivors of acute myocardial infarction (AMI) are at increased risk of subsequent death due to, among others, re-infarction, arrhythmic events, or heart failure. In practically all existing reports [1–3], significantly higher crude follow-up mortality was observed in females compared to males. This has been attributed to the differences in age [4–7], comorbidities [4–6,8], symptom presentation [9–11] and pathophysiology of the underlying coronary artery disease [12–16]. Nevertheless, no solid data exist on the impact of these common sex differences on the post-AMI survival. In particular, data on the importance of the common sex differences are missing in patients treated according to contemporary standards including acute coronary interventions and guideline-based acute pharmacologic treatment.

Whilst the differences between females and males in the survival and severity of AMI have been the topic of a large number of investigations, direct comparisons between large groups of patients of both sexes carefully matched for a number of clinical characteristics are lacking. Such comparisons of case-by-case matched sex groups appear to be the most transparent method for confirming or eliminating the roles that different clinical and pathophysiological factors may play in the sex differences of post-AMI survival and prognosis. Having this in mind, we selected large sex-specific sub-groups of post-AMI patients case-by-case matched for potential confounders that included a majority of the factors that have previously been proposed as the sources of post-AMI sex differences [4–16]. This allowed us to investigate whether these confounders explain the known sex differences in post-AMI survival.

## Methods

Patients of two previously reported prospective cohort studies, namely the ISAR-RISK [17] and ART [18] studies, were investigated. ISAR-RISK was a prospective cohort study aiming to prospectively validate heart rate turbulence in patients who had survived the first 30 days after initial hospitalization for an acute myocardial infarction [19]. ART was a prospective cohort study in the same kind of patients aiming to develop noninvasive risk predictors on the basis of 30-minute high resolution recordings of ECG, blood pressure and respiration [20–24].

ISAR-RISK patients were recruited between January 1996 and April 2000, ART patients between May 2000 and March 2005 at Klinikum rechts der Isar and Deutsches Herzzentrum München, both in Munich (Germany). The last follow-up for both studies was in May 2010. Study patients suffered from AMI within 4 weeks prior to enrolment. AMI was diagnosed if 2 of the following criteria were present: 1) chest pain lasting ≥20 minutes prior to admission, 2) creatine kinase-MB levels above double upper normal limit of our laboratory, 3) ST-Segment elevation of ≥0.1 mV in two or more limb leads and/or ≥0.2 mV in two or more contiguous precordial leads.

Revascularization was performed using percutaneous coronary intervention (PCI), coronary artery bypass grafting (CABG) or thrombolysis. Conservative revascularization therapy was performed in case of severe underlying diseases such as malignancies, diffuse obstructive coronary artery disease (CAD) and refusal of coronary angiography by the patient.

Both source studies were approved by the local ethics committee of Technische Universität München (Munich, Germany). All patients provided oral (ISAR-RISK) or written (ART) informed consent.

### Clinical variables

On hospital admission, heart rate, blood pressure, serum creatinine and cardiac enzymes were measured and a standard 12-lead ECG recorded in all patients. Left ventricular ejection fraction (LVEF) was quantified by either echocardiography or angiography. Atrial fibrillation was diagnosed if present in the resting ECG at admission. Patients were considered to suffer from diabetes if they were already diagnosed and were receiving treatment or if their fasting blood glucose concentration repeatedly exceeded 11 mmol/L during the hospitalization for the index AMI. Patients were considered to suffer from hypertension if they were already diagnosed and were receiving treatment or if their arterial pressure repeatedly exceeded 140/90 mmHg during the hospitalization. Patients were considered to be smokers if they reported smoking at hospital admission.

### Follow-up and endpoints

Patients were followed-up every six months by clinical appointments. In case of non-attendance, patients were contacted personally or their status was assessed through their general practitioner. If the contact was lost, the local population registry provided a new address or confirmed the death of the patient. The primary endpoint was 5-year all-cause mortality.

### Composition of study cohort

A total of 4141 patients of the ISAR-RISK and the ART study were admitted to the hospital with AMI. Of these, 3854 patients survived the first 30 days after hospital admission. We further excluded 14 patients due to incomplete clinical baseline data. In the remaining cohort of 3840 patients, 994 (25.9%) were female and 2846 (74.1%) were male, respectively. In total, 29 (0.8%) patients were lost to follow-up (<1 year) and were censored at their last attended clinical appointment.

### Statistics

Cohorts of 1:1 matched female and male patients were selected among all patients of the dataset to account for differences in known possible confounders of follow-up mortality allowing a comparison of survival times for females and males who are similar in relevant patient characteristics. For each female patient, a corresponding male patient was selected with corresponding age, LVEF, hypertension, diabetes mellitus, smoking status, non-sinus rhythm, previous AMI, and the category of revascularization therapy (i.e. PCI, CABG, thrombolysis, or “no intervention” which included patients without obstructive CAD and those treated conservatively in the presence of severe underlying disease, diffuse obstructive coronary artery disease, or coronary angiography refusal).

Only patients within the convex hull of the variables for females and males were considered to ensure similarity between matched female and male patients [25]. Within these patients, an exact match of the categorical variables (hypertension, diabetes mellitus, smoking status, non-sinus rhythm, previous AMI, and revascularization therapy) was performed. For quantitative measures, i.e. age and LVEF, a nearest-neighbor matching based on the propensity score [26] was used to find the best control match. Matching was performed using the R package “MatchIt” [27].

Continuous variables are presented as mean ± standard deviation (SD). Categorical data are presented as absolute frequencies and percentages. Group means were compared by two-tailed t-tests using Welch’s adaption for different group variances. Frequencies were compared by Chi-Square tests of Fisher exact tests. Survival curves were estimated by the Kaplan–Meier method and compared using the log-rank test. Confidence bands of the Kaplan-Meier curves were calculated using the bootstrap technique with 10,000 repetitions. Cox regression models were fit to the data to estimate hazard ratios with corresponding 95% confidence intervals (CI). Survival times were compared between females and males in the whole study cohort and in a cohort matched with regard to relevant confounders.

Additional survival analyses were performed to compare mortality risks for different time intervals. Specifically, separate analyses considered the sex survival differences during the first year after the index AMI, and the sex survival differences among patients who survived for at least one year after the index AMI.

A p-value of <0.05 was considered as statistically significant. IBM SPSS Statistics 22.0 (IBM SPSS, Inc.) and R 3.2.2 (R Foundation for Statistical Computing, Vienna, Austria) were used for the statistical analyses.

## Results

### Complete cohort

Table 1 shows the clinical characteristics of the complete cohort at baseline hospitalization. Females were older and suffered more frequently from hypertension and diabetes mellitus. They were less often active smokers. A previous AMI in the past medical history was more often among males. Fewer females than males were in sinus rhythm. Males showed a significantly worse renal function than females. CK-levels at admission were significantly higher in males than in females. There were no significant LVEF differences between sexes.

**Table 1:** Patient characteristics in the complete cohort (n = 3,840) at baseline hospitalization.

	Females n = 994	Males n = 2846	P
**Clinical data**			
Age (years), mean (SD)	68.7 (11.9)	61.0 (12.2)	<0.001
Hypertension, n (%)	745 (74.9)	1853 (65.1)	<0.001
Diabetes mellitus, n (%)	255 (25.7)	549 (19.3)	<0.001
Smokers, n (%)	272 (27.4)	1542 (54.2)	<0.001
Creatinine(mg/dl), mean (SD)	1.2 (0.5)	1.3 (0.2)	<0.001
Previous AMI, n (%)	102 (10.3)	386 (13.6)	0.008
Non-SR, n (%)	79 (7.9)	166 (5.8)	0.023
CK max (U/l), mean (SD)	1526 (1583)	2017 (2385)	<0.001
LVEF (%),mean (SD)	52.6 (13.4)	52.0 (13.0)	0.177
Coronary angiography, n (%)	986 (99.2)	2836 (99.6)	0.125
Non-obstructive CAD, n (%)	52 (5.2)	69 (2.4)	<0.001
One-vessel CAD, n (%)	357 (35.9)	924 (32.5)	0.052
Two-vessel CAD, n (%)	258 (26.0)	790 (27.8)	0.291
Three-vessel CAD, n (%)	327 (32.9)	1063 (37.4)	0.013
**Therapy**			
PCI, n (%)	852 (85.7)	2589 (91.0)	<0.001
CABG, n (%)	28 (2.8)	84 (3.0)	0.914
Thrombolysis n (%)	24 (2.4)	60 (2.1)	0.658
Conservative, n (%)	90 (9.1)	113 (4.0)	<0.001
ASS, n (%)	964 (97.0)	2759 (96.9)	1.000
Betablockers, n (%)	921 (92.7)	2609 (91.7)	0.362
ACE inhibitors, n (%)	880 (88.5)	2550 (89.6)	0.379
Statins, n (%)	829 (83.4)	2414 (81.0)	0.311
Diuretics, n (%)	472 (47.5)	1195 (42.0)	0.003
**Mortality**			
5-year all-cause, n (%)	175 (17.6)	337 (11.8)	<0.0001

ACE inhibitor: angiotensin-converting enzyme inhibitor, AMI: myocardial infarction, ASS: acetylsalicylic acid, CABG: coronary artery bypass grafting, CAD: coronary artery disease, CK max: maximal level of creatine kinase, LVEF: left ventricular ejection fraction, PCI: percutaneous coronary intervention, SD: standard deviation, SR: sinus rhythm

Coronary angiography was performed in 99.2% and 99.6% of female and male patients, respectively. A non-obstructive CAD was significantly more often noted in females than in males. Accordingly, females were more frequently treated conservatively regarding PCI revascularization. Males showed a significantly higher rate of three-vessel CAD. Thrombolysis and CABG were infrequent in both groups without significant differences.

Drug therapy after AMI with acetylsalicylic acid (ASS), betablockers, angiotensin-converting enzyme inhibitors (ACE-inhibitors) and statins did not differ significantly between sexes. The use of diuretics was more frequent in females.

**Fig 1:**
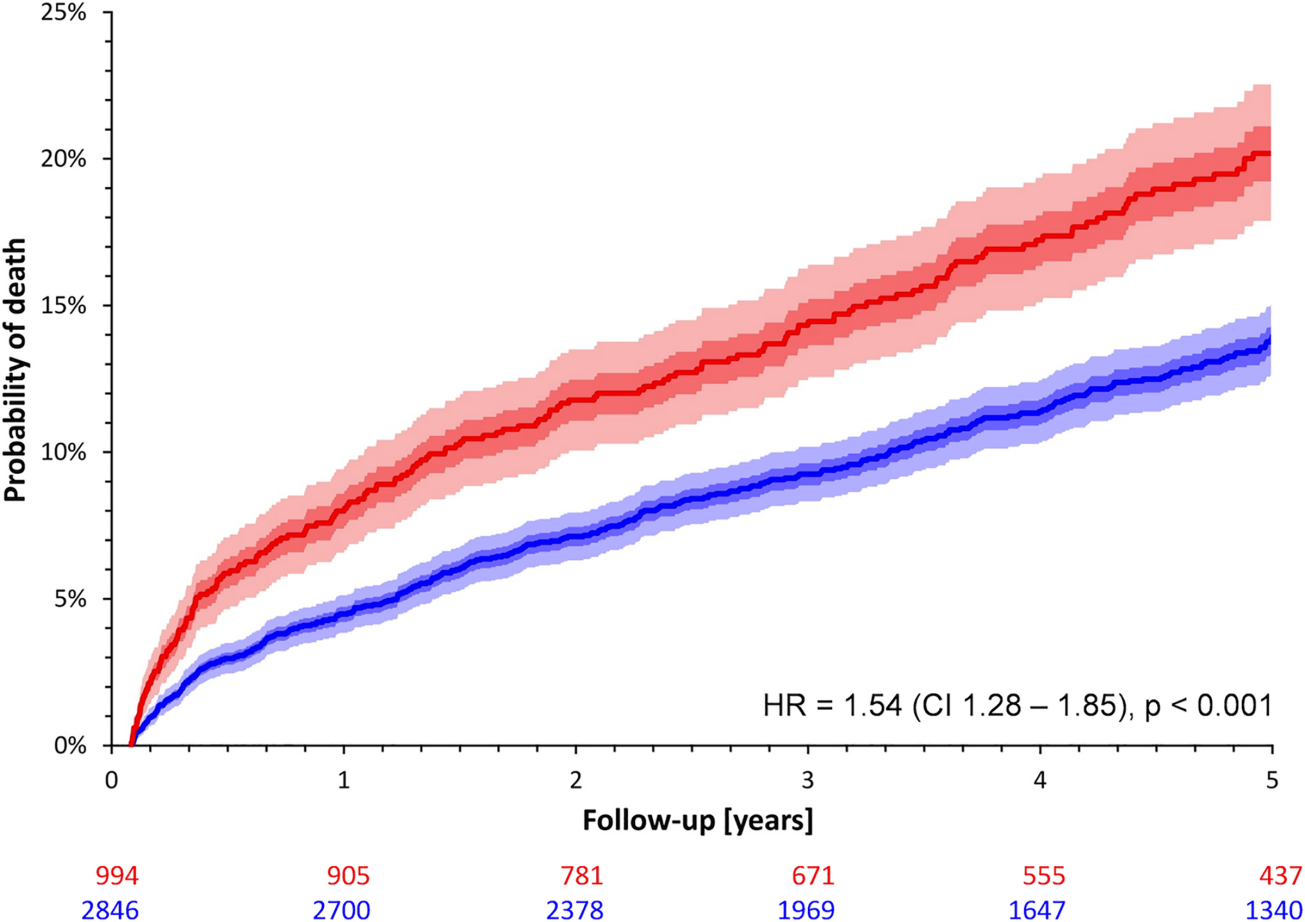
Probabilities of death stratified by sex in the entire study cohort. Red and blue lines and bands correspond to females and males, respectively. The dark shaded and light shaded areas correspond to inter-quartile bands and 90% confidence bands of the Kaplan-Meier probability curves, respectively. Numbers of patients at risk are shown below the graph in colors corresponding to the probability curves. CI– 95% confidence interval; HR–hazard ratio of females vs. males.

The probability of death of any cause was significantly higher in females during the five years of follow-up (17.6% vs. 11.8%; HR for female sex 1.54; CI 1.28–1.85; p<0.0001; Fig 1).

### Matched cohort

The matched cohort included 1604 patients; 802 patients of either sex. Table 2 presents the clinical characteristics of the matched cohort. Clinical parameters regarding cardiovascular risk factors and revascularization therapy were equally distributed between the sexes with age nearly balanced (67.5 years in females versus 67.0 years in males, p = 0.153). Serum creatinine was significantly higher in males than in females. No statistically significant differences were observed with respect of pharmaceutical therapy with the exception of ACE inhibitors which were less often administered in females (89.7% versus 92.8%, p = 0.034). Almost all patients underwent coronary angiography (≥99.5%) in both sexes. The prevalence of non-obstructive CAD was non-significantly more frequent in females (3.4% in females versus 2.0% in males, p = 0.122). Prevalence of one-vessel CAD was significantly more often in females, a three-vessel CAD predominated in males.

**Table 2:** Clinical characteristics in the matched cohort (n = 1,604).

	Females n = 802	Males n = 802	P
**Clinical data**			
Age (years), SD	67.5 (11.1)	67.0 (10.7)	0.153
Hypertension, n (%)	613 (76.4)	613 (76.4)	1
Diabetes mellitus, n (%)	192 (23.9)	192 (23.9)	1
Smokers, n (%)	226 (28.2)	226 (28.2)	1
Creatinine(mg/dl),mean (SD)	1.1 (0.5)	1.3 (0.4)	<0.001
Previous AMI, n (%)	50 (6.2)	50 (6.2)	1
Non-SR, n (%)	34 (4.2)	34 (4.2)	1
CK max (U/l), mean (SD)	1580 (1612)	1819 (2232)	0.008
LVEF (%), SD	53.4 (11.7)	53.4 (11.3)	0.939
Coronary angiography, n (%)	798 (99.5)	799 (99.6)	1
Non-obstructive CAD, n (%)	27 (3.4)	16 (2.0)	0.122
One-vessel CAD, n (%)	310 (38.7)	239 (29.8)	<0.001
Two-vessel CAD, n (%)	222 (27.7)	228 (28.4)	0.781
Three-vessel CAD, n (%)	243 (30.3)	319 (39.8)	<0.001
**Therapy**			
PCI, n (%)	758 (94.5)	761(94.9)	0.824
CABG, n (%)	6 (0.7)	7 (0.9)	1
Thrombolysis, n (%)	3 (0.4)	3 (0.4)	1
Conservative, n (%)	35 (4.4)	31 (3.9)	0.706
ASS, n (%)	781 (97.4)	784 (97.8)	0.746
Betablockers, n (%)	753 (93.9)	741 (92.4)	0.277
ACE inhibitors, n (%)	719 (89.7)	744 (92.8)	0.034
Statins, n (%)	677 (84.4)	697 (86.9)	0.176
Diuretics, n (%)	361 (45.0)	348 (43.4)	0.546
**Mortality**			
5-year all-cause, n (%)	109 (13.6)	94 (11.7)	0.293

Matching was performed according to the composite of age, LVEF, hypertension, diabetes mellitus, smoking status, non-sinus rhythm, previous AMI and revascularization therapy. ACE inhibitor: angiotensin-converting enzyme inhibitor, AMI: myocardial infarction, ASS: acetylsalicylic acid, CABG: coronary artery bypass grafting, CAD: coronary artery disease, CK max: maximal level of creatine kinase, LVEF: left ventricular ejection fraction, PCI: percutaneous coronary intervention, SD: standard deviation, SR: sinus rhythm

**Fig 2:**
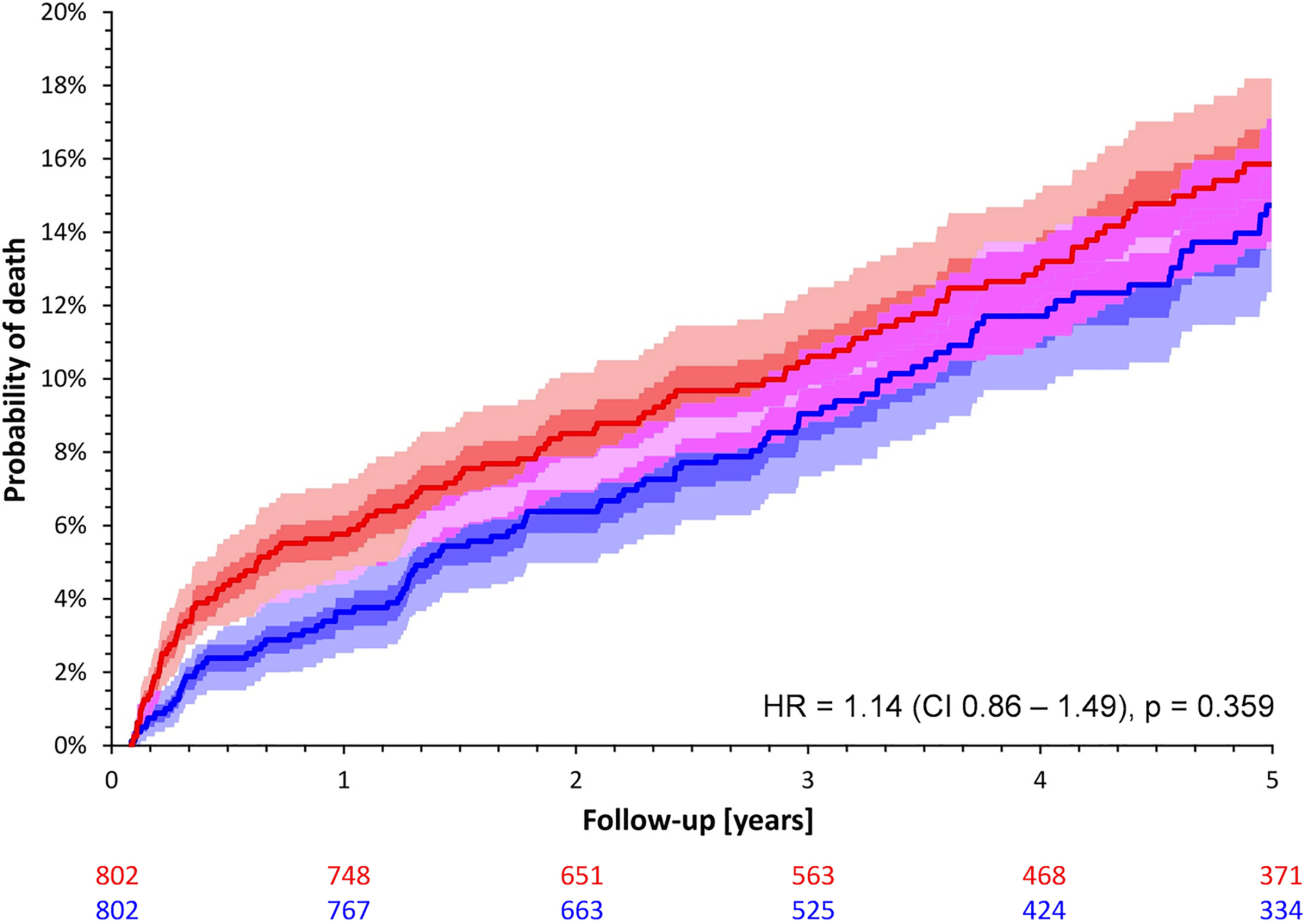
Probabilities of death stratified by sex in the cohort of matched patients. Red and blue lines and bands correspond to females and males, respectively. The dark shaded and light shaded areas correspond to inter-quartile bands and 90% confidence bands of the Kaplan-Meier probability curves, respectively. Light violet areas show the overlap of the 90% confidence bands, dark violet areas show the overlap of the inter-quartile bands of one of the probability curves with the 90% confidence band of the other curve. Numbers of patients at risk are shown below the graph in colors corresponding to the probability curves. CI– 95% confidence interval; HR–hazard ratio of females vs. males.

**Fig 3:**
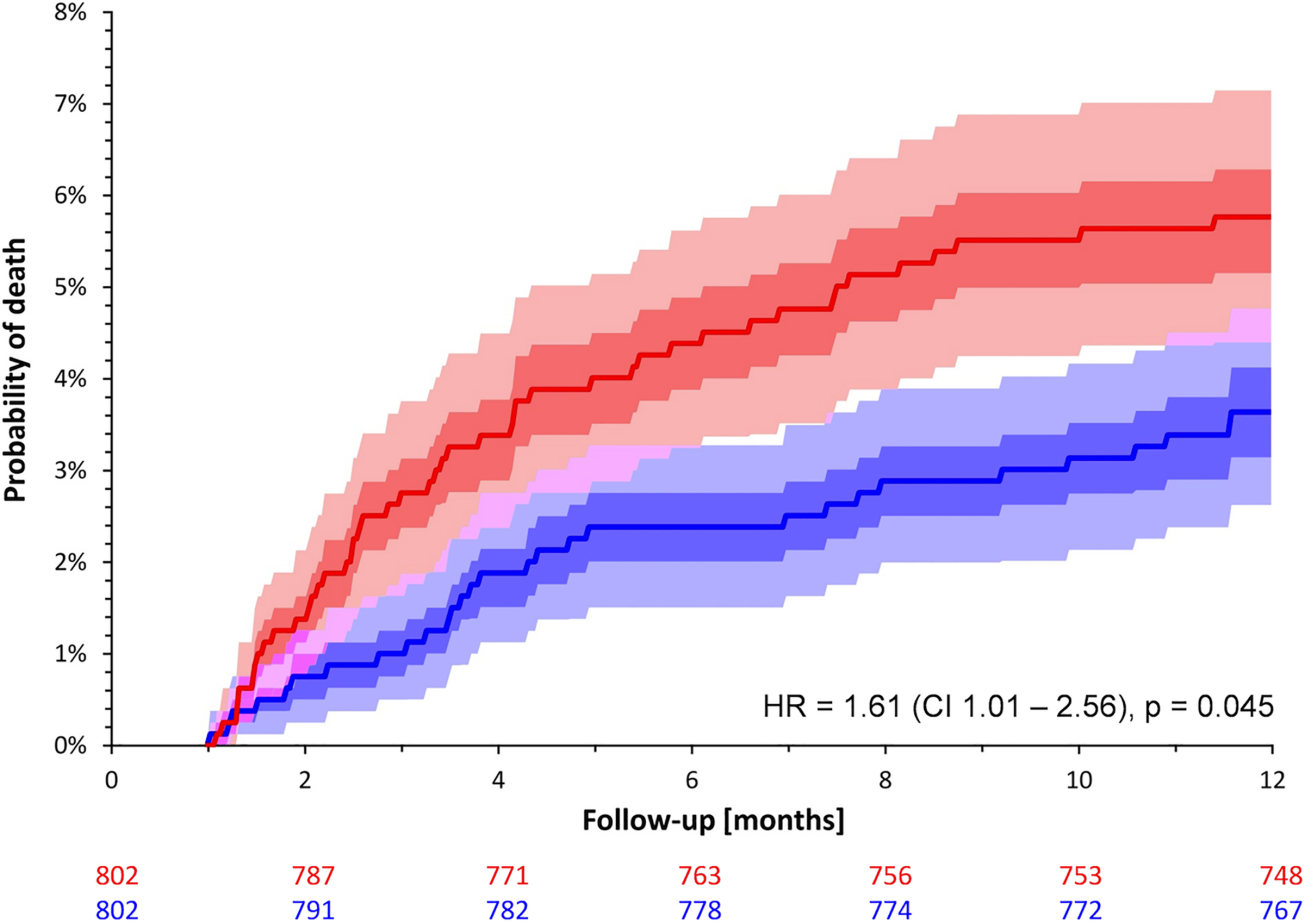
Probabilities of death stratified by sex in the sex-matched cohort during the first year after the index infarction. See Fig 2 for details.

**Fig 4:**
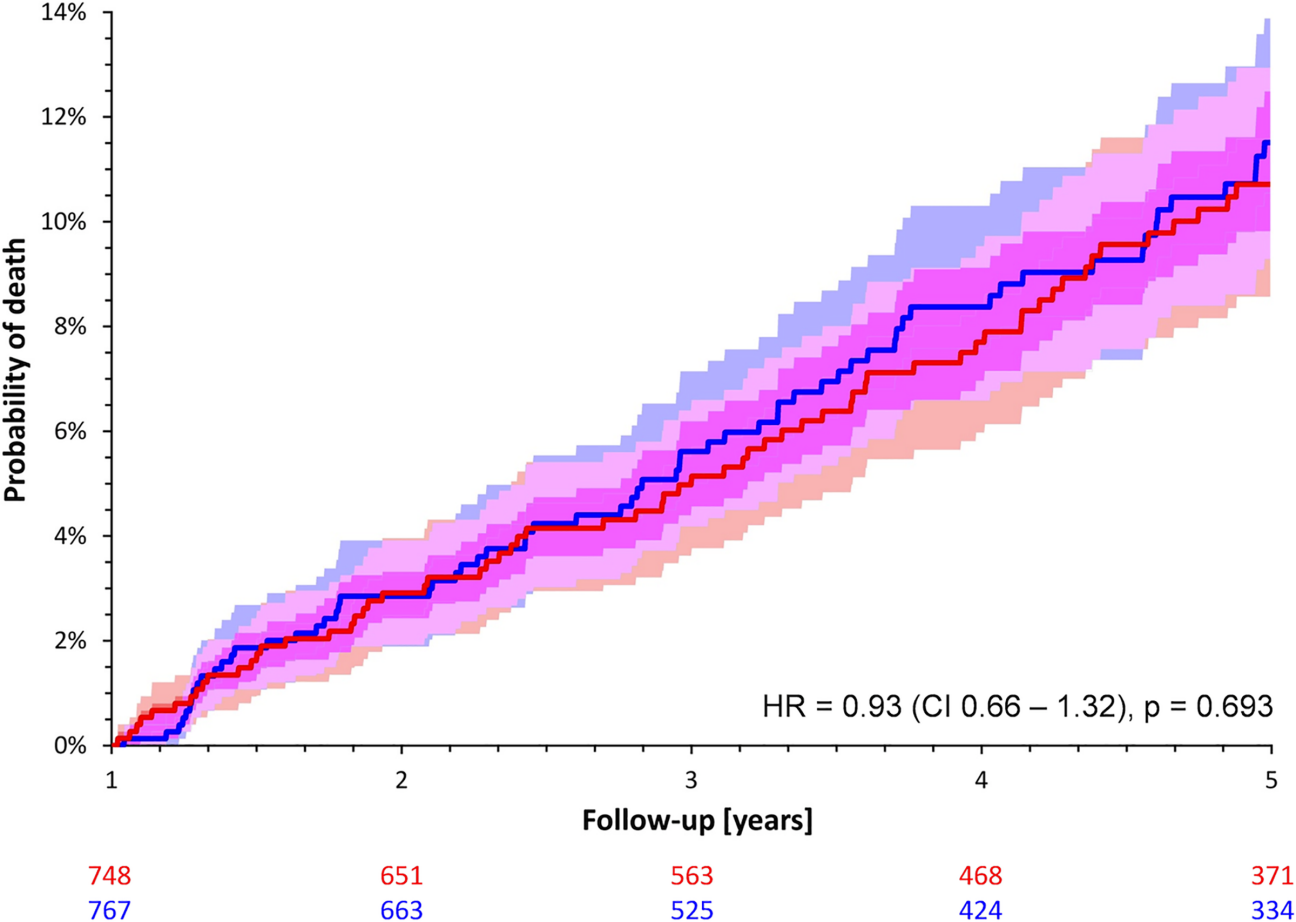
Probabilities of death stratified by sex in the sex-matched cohort between the second and fifth year after the index infarction. See Fig 2 for details.

Over the entire follow-up period of five years, the mortality rates of female and male patients of the matched cohort did not differ significantly although a trend towards a poorer survival in females was observed (13.6% versus 11.7%; HR for female sex 1.14; CI 0.86–1.49; p = 0.359; Fig 2). However, when restricting the analysis to the first year of follow-up, female patients had a significantly worse outcome (mortality of 5.7% in females versus 3.6% in males; HR 1.61; CI 1.01–2.56; p = 0.045; Fig 3). The mortality between years 2 and 5 was practically identical between both sexes (8.1% in men versus 7.9% in women; HR 0.93; CI 0.66–1.32; p = 0.693; Fig 4).

### Comparison between unmatched and matched females

No matching male counterpart was found for 192 females (19.3% of all female patients), because it was impossible to find absolute correspondence in all of the matching criteria. Table 3 shows the comparison between unmatched and matched females. There were substantial and statistically significant differences in many of the characteristics with the unmatched females being markedly older, suffering more frequently from diabetes mellitus and hypertension and showing a significantly higher rate of non-obstructive CAD (13.0% in unmatched females versus 3.4% in matched females, p<0.001). Rates of revascularization by PCI were strikingly less frequent in unmatched females compared to matched females (49.0% versus 94.5%, p<0.001) and unmatched females were more often treated conservatively (28.6% versus 4.4%, p<0.001).

**Table 3:** Patient characteristics of unmatched and matched females (n = 994).

	Unmatched Females n = 192	Matched Females n = 802	P
**Clinical data**			
Age (years), mean (SD)	73.7 (13.9)	67.5 (11.1)	<0.001
Hypertension, n (%)	132 (68.8)	613 (76.4)	0.034
Diabetes mellitus, n (%)	63 (32.8)	192 (23.9)	0.015
Smokers, n (%)	46 (24.0)	226 (28.2)	0.276
Creatinine(mg/dl), mean (SD)	1.21 (0.52)	1.10 (0.52)	0.005
Previous AMI, n (%)	52 (27.1)	50 (6.2)	<0.001
Non-SR, n (%)	45 (23.4)	34 (4.2)	<0.001
CK max (U/l), mean (SD)	1300 (1443)	1580 (1612)	0.011
LVEF (%),mean (SD)	49.3 (18.8)	53.4 (11.7)	<0.001
Coronary angiography, n (%)	188 (97.9)	798 (99.5)	0.079
Non-obstructive CAD, n (%)	25 (13.0)	27 (3.4)	<0.001
One-vessel CAD, n (%)	47 (24.5)	310 (38.7)	<0.001
Two-vessel CAD, n (%)	36 (18.8)	222 (27.7)	0.015
Three-vessel CAD, n (%)	84 (43.8)	243 (30.3)	0.001
**Therapy**			
PCI, n (%)	94 (49.0)	758 (94.5)	<0.001
CABG, n (%)	22 (11.5)	6 (0.7)	<0.001
Thrombolysis n (%)	21 (10.9)	3 (0.4)	<0.001
Conservative, n (%)	55 (28.6)	35 (4.4)	<0.001
ASS, n (%)	183 (95.3)	781 (97.4)	0.204
Betablockers, n (%)	168 (87.5)	753 (93.9)	0.004
ACE inhibitors, n (%)	161 (83.9)	719 (89.7)	0.033
Statins, n (%)	152 (79.2)	677 (84.4)	0.099
Diuretics, n (%)	111 (57.8)	361 (45.0)	0.002
**Mortality**			
5-year all-cause, n (%)	66 (34.4)	109 (13.6)	<0.001

ACE inhibitor: angiotensin-converting enzyme inhibitor, AMI: myocardial infarction, ASS: acetylsalicylic acid, CABG: coronary artery bypass grafting, CAD: coronary artery disease, CK max: maximal level of creatine kinase, LVEF: left ventricular ejection fraction, PCI: percutaneous coronary intervention, SD: standard deviation, SR: sinus rhythm

**Fig 5:**
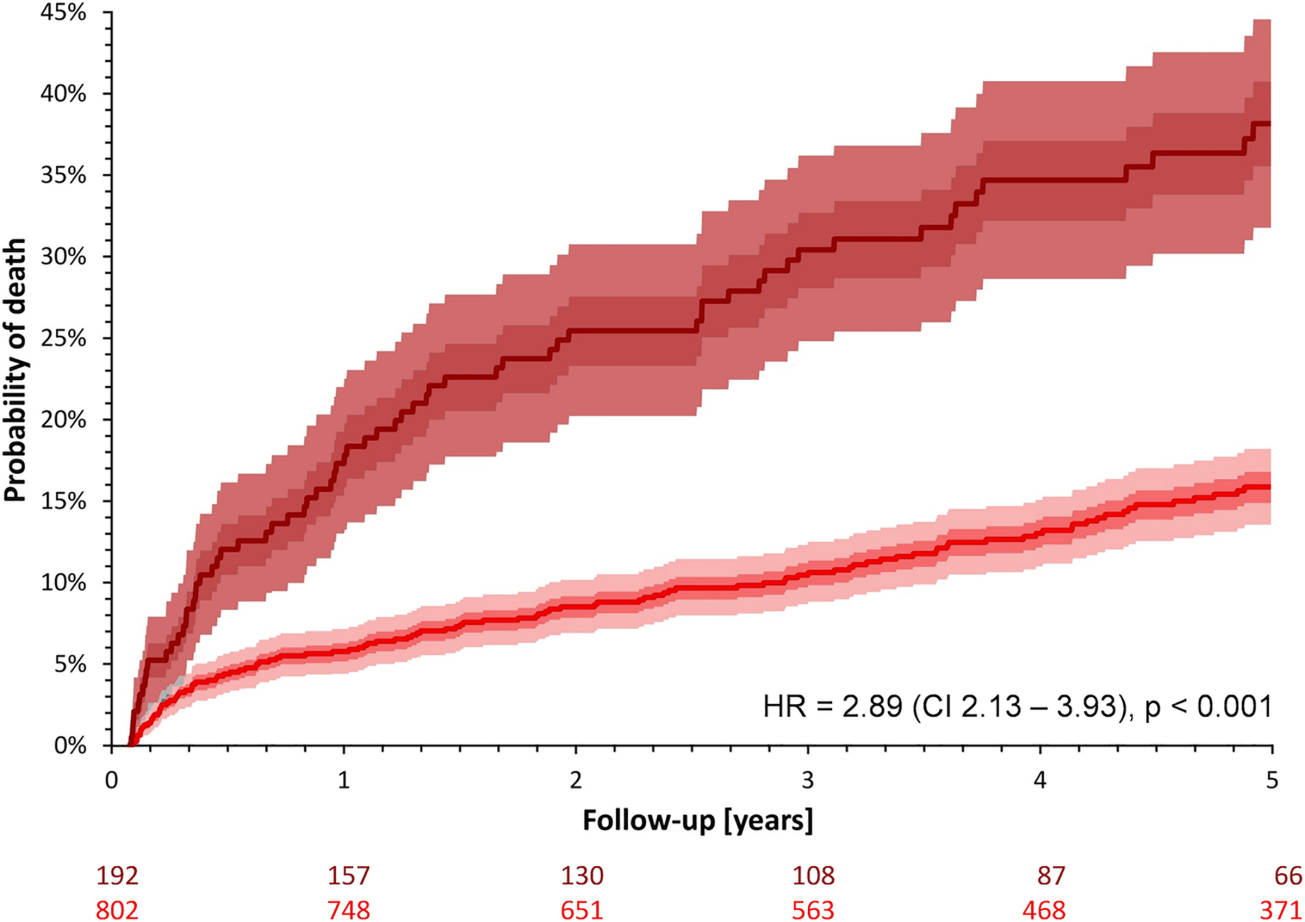
Probabilities of death comparing females included and not included in the sex-matched cohort. Red and brown lines and bands correspond to females included (n = 802) and not included (n = 192) in the sex-matched cohort, respectively. The dark shaded and light shaded areas correspond to inter-quartile bands and 90% confidence bands of the Kaplan-Meier probability curves, respectively. Light grey areas show the overlap of the 90% confidence bands, dark grey areas show the overlap of the inter-quartile bands of one of the probability curves with the 90% confidence band of the other curve. Numbers of patients at risk are shown below the graph in colors corresponding to the probability curves. CI– 95% confidence interval; HR–hazard ratio of females not included vs. included in the sex-matched cohort.

Not surprisingly, the survival of the unmatched females was also substantially poorer compared to matched females (5-year mortality of 34.4% versus 13.6%; HR = 2.89; CI 2.13–3.93; p = <0.0001; Fig 5).

### Comparison between unmatched and matched males

Contrary to females, comparison between unmatched and matched males reveals a significant younger age and a lower prevalence of hypertension and diabetes mellitus for the unmatched patients. There was no statistical significant difference regarding the non-obstructive or obstructive CAD between these groups. Table 4 shows the comparison between unmatched and matched males.

**Table 4:** Patient characteristics of unmatched and matched males (n = 2846).

	Unmatched Males n = 2044	Matched Males n = 802	P
**Clinical data**			
Age (years), mean (SD)	58.7 (11.9)	67.0 (10.7)	<0.001
Hypertension, n (%)	1240 (60.6)	613 (76.4)	<0.001
Diabetes mellitus, n (%)	357 (17.5)	192 (23.9)	<0.001
Smokers, n (%)	1316 (64.4)	226 (28.2)	<0.001
Creatinine(mg/dl), mean (SD)	1.22 (0.42)	1.25 (0.41)	0.008
Previous AMI, n (%)	336 (16.4)	50 (6.2)	<0.001
Non-SR, n (%)	132 (6.5)	34 (4.2)	0.029
CK max (U/l), mean (SD)	2095 (2438)	1819 (2232)	0.016
LVEF (%),mean (SD)	51.5 (13.6)	53.4 (11.3)	0.008
Coronary angiography, n (%)	2037 (99.7)	799 (99.6)	1.0
Non-obstructive CAD, n (%)	53 (2.6)	16 (2.0)	0.425
One-vessel CAD, n (%)	685 (33.5)	239 (29.8)	0.063
Two-vessel CAD, n (%)	562 (27.5)	228 (28.4)	0.650
Three-vessel CAD, n (%)	744 (36.4)	319 (39.8)	0.103
**Therapy**			
PCI, n (%)	1828 (89.4)	761(94.9)	<0.001
CABG, n (%)	77 (3.8)	7 (0.9)	<0.001
Thrombolysis n (%)	57 (2.8)	3 (0.4)	<0.001
Conservative, n (%)	82 (4.0)	31 (3.9)	0.942
ASS, n (%)	1975 (96.6)	784 (97.8)	0.145
Betablockers, n (%)	1868 (91.4)	741 (92.4)	0.425
ACE inhibitors, n (%)	1806 (88.4)	744 (92.8)	0.001
Statins, n (%)	1717 (84.0)	697 (86.9)	0.059
Diuretics, n (%)	847 (41.4)	348 (43.4)	0.364
**Mortality**			
5-year all-cause, n (%)	243 (11.9)	94 (11.7)	0.952

ACE inhibitor: angiotensin-converting enzyme inhibitor, AMI: myocardial infarction, ASS: acetylsalicylic acid, CABG: coronary artery bypass grafting, CAD: coronary artery disease, CK max: maximal level of creatine kinase, LVEF: left ventricular ejection fraction, PCI: percutaneous coronary intervention, SD: standard deviation, SR: sinus rhythm

**Fig 6:**
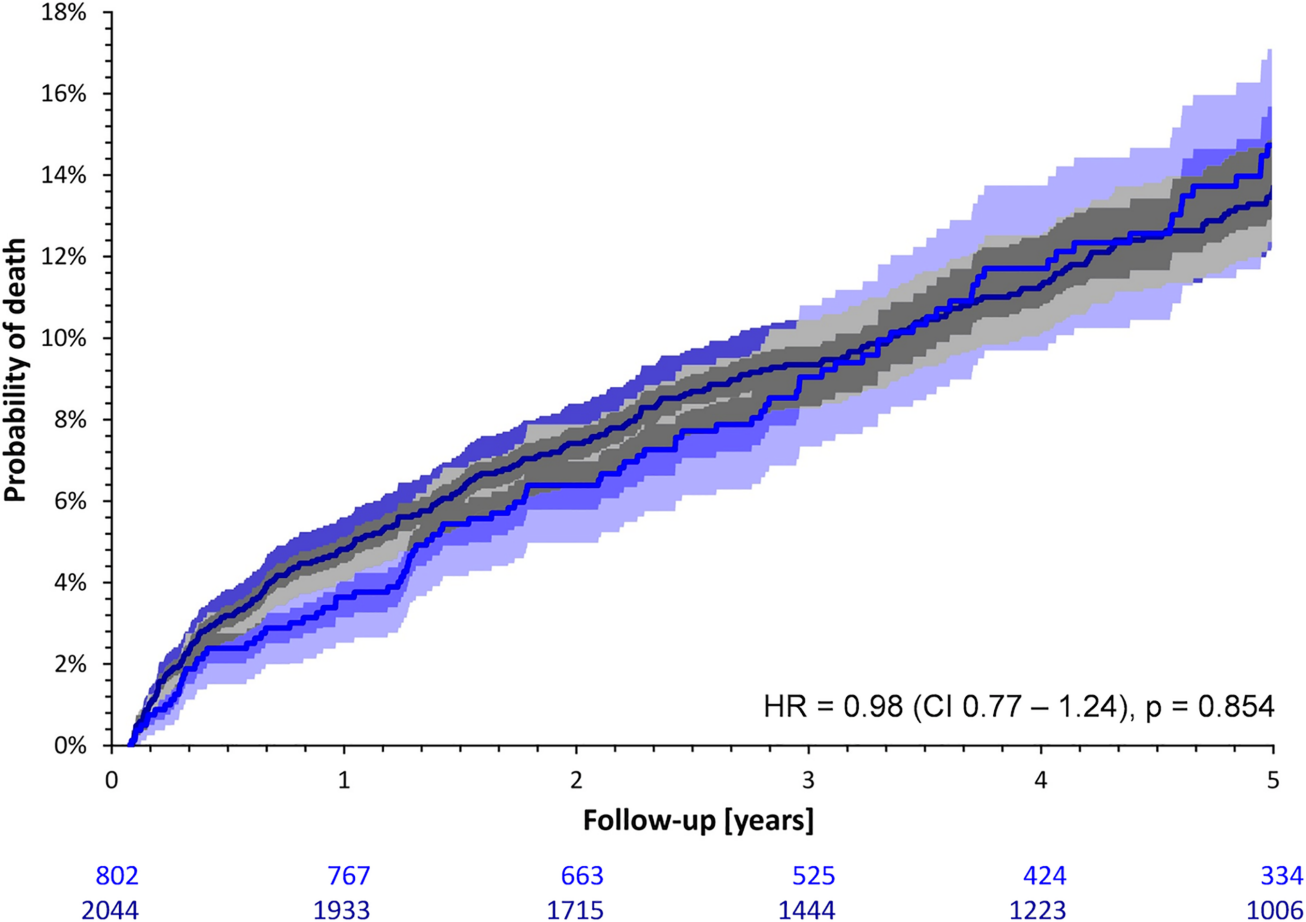
Probabilities of death comparing males included and not included in the sex-matched cohort. Light blue and dark blue lines and bands correspond to males included (n = 802) and not included (n = 2044) in the sex-matched cohort, respectively. Further explanations correspond to Fig 5. HR–hazard ratio of men not included vs. included in the sex-matched cohort.

In contrast to females, survival rates between unmatched and matched males were similar (11.9% versus 11.7%; HR = 0.98; CI 0.77–1.24; p = 0.854; Fig 6). Note that also the survival rates between matched and unmatched males were also similar during the first year after the index AMI (p = 0.172, detailed data not shown).

## Discussion

In agreement with previous studies [1,4,28–31], female post-infarction patients included in our prospective cohort study had a significantly higher mortality risk compared to males. Our data agree with previous explanations of this difference by female patients being older [4–7], having a higher prevalence of comorbidities such as hypertension and diabetes [4–6,8] and of non-obstructive CAD [12–16], being subject to a less invasive diagnostic [6,32] or therapeutic approach [6,33–36] as well as by the composite of different variables [37]. Indeed, comparison of Figs 5 and 6 shows that while the subgroup of sex-matched males was, in terms of post-AMI survival, representative of all the males, this was not the case for females. Not only the previously reported risk factors but also their clinically more severe combinations are more frequent among females compare to males.

Aim of the matching analysis was to generate sets of females and males that were comparable regarding relevant risk factors in order to compare the sex-specific risk in a fair and systematic manner.

Importantly—as a result of the strict matching process—the oldest and sickest females were excluded from the matched cohort. Unmatched females had a higher risk factor burden, suffered more often from non-obstructive CAD, and had an almost 3-fold increase in 5-year mortality compared to those for whom a corresponding male patient was found.

In a matched cohort with the potentially influencing variables balanced, all-cause mortality during 5-year follow-up did not differ significantly between sexes over the whole 5-year follow-up period. However, we observed a significantly increased mortality risk in females during the first year of follow-up (HR 1.61; p = 0.045). Since the matched criteria can be excluded as reasons for the observed sex-specific mortality difference, other clinical and non-clinical aspects need to be considered as possible explanations for the observed mortality difference.

From the clinical point of view, factors such as time to PCI or thrombolysis as well as different pathologies leading to infarction such as dynamic obstruction, endothelial dysfunction or stress cardiomyopathy need to be taken into account. We could not evaluate all these possibilities in our matching analysis since the matching sub-groups would become too small and non-representative.

When recruitment for the ISAR-RISK [17] and ART [18] studies was initiated, distinctive knowledge about the mortality impact of the symptoms-to-balloon time [38–40] was only evolving. Consequently, these data were not routinely collected. We cannot exclude their influence on the observed mortality difference. However, we assume that treatment in females could not have been significantly delayed, as higher ischemic burden would have led to significantly increased CK-levels or impaired left ventricular function compared to males, which was not the case in the matched cohort.

Former studies reported higher renal dysfunction in female ST-elevation myocardial infarction (STEMI) patients and detected equal increment of mortality in both sexes in case of renal impairment [41,42]. In our cohort, males showed a decreased renal function compared to females in both the total and matched cohort. Still, female mortality exceeded that of males. This observation is even more surprising in this context.

Leaving the clinical aspects aside, a number of studies revealed non-clinical sex-specific factors such as socioeconomic status or depression contributing to worse outcome in female post-AMI patients [43–45]. Compared to males, female AMI survivors present more often with psychological stress. They have lower scores on mental health status [46], show a greater functional decline and poorer health-related quality of life [47–53] and also suffer more frequently from depression [54]. At the same time, psychosocial factors are known to play an important role in post-AMI recovery and to predict 1-year cardiac mortality, independent of the severity of cardiac disease [45,46,51]. Correspondingly, family life status is known to influence post-AMI survival [45,55,56]. As we did not have the data to assess social status, marital status, quality of life or presence of depressive symptoms in this cohort, we cannot exclude these non-clinical factors from a partial influence on the detected mortality difference during the first year after AMI.

Equally importantly, autonomic function might play a role in respect of post-AMI mortality rates independently of clinical and social sex-specific differences. Measures of autonomic regulation processes have been proven as independent and useful risk stratification parameters [57]. However, studies investigating the impact of sex-dependent differences in cardiac autonomous nervous system on the outcome after AMI are rare and have been so far inconclusive.

Finally, the possibility that the early divergence in mortality risk in our matched cohort is due to a play of chance cannot be entirely excluded.

Despite the number of the confounders that we could not have included in the matching process, our observations have important clinical implications. The excess mortality among females in the matched subgroups was observed during first year after the index AMI. Female AMI patients should therefore be followed more actively in the early post-AMI stages with more frequent follow-up clinical assessments at least in the first year after the event. Attention should be given to psychological and psycho-social factors in female patients with appropriate referrals to relevant specialists. Psychological support through specialists’ sessions and/or patient self-supporting groups for inter-patient communication and discussion should be considered.

Since the confounders that we have used for matching the sex groups are frequently used in guidelines and consensus proposals for post-AMI clinical care, our results also suggest that it is not fully appropriate to rely on evidence obtained in studies of predominantly male patients when treating female patients. Separate studies in female patients are needed to confirm whether the male-based evidence is appropriate or whether different guideline criteria are needed for both sexes.

### Limitations

Several limitations of our study need to be considered. In the original ISAR-Risk and ART studies, no detailed information was collected about clinical data regarding symptoms-to-balloon-time or coronary pathology including endothelial dysfunction causing the myocardial infarction. Apart from smoking, we are unable to include any data on pre-AMI lifestyles. Also, socioeconomic aspects, family support and the prevalence of depression were not assessed. We can only assume that these factors had substantial impact on mortality rates in the matched cohort, but cannot exclude other so far unknown factors.

Although the identification of the 1:1 matching groups was performed using previously proposed factors contributing to the survival differences, some differences between the matched groups could not have been eliminated. Not only were the women in the case-matched analysis less frequently treated with ACE inhibitors, they were also tiny bit older and suffered more frequently from a non-obstructive CAD. It was impossible to include pharmacological treatments (including that by ACE inhibitors) into the matching procedure, as the matched groups would have been too small and non-representative.

As risk profiles between sexes differed such substantially—especially regarding age—it was impossible to find a female-male match in one fifth of female patients. More liberal matching criteria could have been considered but this would have led to groups differing relevantly in important confounding factors. Since the number of females without an adequate match was already substantial when using 1:1 matching, we decided against a more flexible form of matching, e.g. 1:2 or 1:3, as the number of unmatched females would have been substantially increased.

Our data are based on patient recruitment between 1996 and 2005. Both medical therapy and revascularization techniques are improving over time. Particularly, the use of drug eluting stents and dual antiplatelet therapy evolved since the initial recruitment. However, as far as these clinical advances are concerned, we can safely assume that at each time point, they would have been applied to female and male patients similarly. It is therefore not likely that clinical advances over study duration impacted on the mortality differences. Finally, the retrospective nature of the analysis of prospectively-collected data needs to be acknowledged.

## Conclusion

In this study, a typical cohort of AMI survivors was investigated with focus on sex-specific mortality. After matching females with 1:1 corresponding males of the same age, LVEF, cardiovascular risk factors and revascularization therapy, sex differences in post AMI survival could not be fully eliminated, particularly during the first year after the infarction. This finding is rather surprising and of clinical relevance.

Remaining excess female mortality might be, at least partly, explained by different pathophysiology of CAD in females and reduced applicability of invasive therapeutic approaches. Non-clinical variables including quality of life, family status and negative psychosocial responses triggered by AMI have to be considered. Nevertheless, we believe that our findings should encourage physicians to attend female AMI survivors with increased attention to prevent worse outcome in the future. In particular, our results appear to suggest that female post-AMI patients should be followed more actively with more frequent clinical and psychological assessments and support during at least the first year after AMI.

## Supporting information

S1 Table Study raw data. PtNo: Patient Number, AMI: acute myocardial infarction, SR: sinus rhythm, CKmax: maximal level of creatine kinase, LVEF: left ventricular ejection fraction, CAD: coronary artery disease, ASS: acetylsalicylic acid, ACE_inhibitors: angiotensin-converting enzyme inhibitor, CSE_inhibitors: cholesterol synthesis enzyme inhibitor, fup_time: follow-up time.
(XLSX)
